# Flexible Biobased Thermosets from Epoxidized Plant Oils: A Study of Aliphatic Cross-Linking Agents

**DOI:** 10.1021/acsapm.4c03944

**Published:** 2025-03-12

**Authors:** Jan Janesch, Axel Solt-Rindler, Lara Dumschat, Oliver Vay, Alice Mija, Wolfgang Gindl-Altmutter, Thomas Rosenau, Wolfgang Raffeiner, Christian Hansmann

**Affiliations:** 1 Institute of Wood Technology and Renewable Materials, Department of Natural Sciences and Sustainable Resources, BOKU—University of Natural Resources and Life Sciences, Vienna, Konrad-Lorenz-Straße 24, 3430 Tulln, Austria; 2 Institute of Chemistry of Renewable Resources, Department of Natural Sciences and Sustainable Resources, BOKU—University of Natural Resources and Life Sciences, Vienna, Konrad-Lorenz-Straße 24, 3430 Tulln, Austria; 3 Wood K plus-Competence Centre for Wood Composites & Wood Chemistry, Kompetenzzentrum Holz GmbH, Altenberger Straße 69, 4040 Linz, Austria; 4 Institute of Chemistry of Nice, Université Côte d’Azur, UMR CNRS 7272, 06108 Nice CEDEX 2, France; 5 Sozialgenossenschaft “Lebenswertes Ulten”, Gewerbezone Schmiedhof 349, 39016 St. Walburg, Italy

**Keywords:** Epoxidized linseed oil, biobased epoxy, flexible epoxy, green thermoset, aliphatic amine hardener, epoxidized plant oil

## Abstract

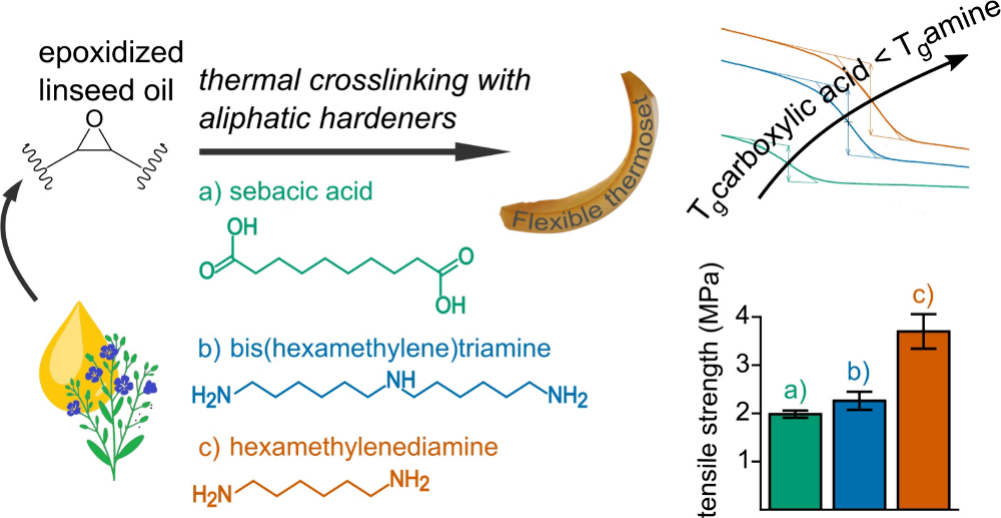

This study investigates the preparation of flexible biobased thermosets by cross-linking epoxidized linseed oil (ELO) with three different hardeners: hexamethylene diamine (HMDA), bis(hexamethylene)triamine (BHMT), and sebacic acid. In a comparative analysis of amine and carboxylic acid cross-linkers, the mechanical, thermal, and chemical properties of the resulting thermosets were evaluated using Fourier transform infrared (FT-IR) spectroscopy, differential scanning calorimetry (DSC), dynamic mechanical analysis (DMA), thermogravimetric analysis (TGA), and tensile testing. FT-IR spectroscopy revealed the formation of an amide network in samples cured by using amine hardeners. HMDA and BHMT provided superior mechanical properties, with tensile strengths of 3.7 MPa and 2.3 MPa, respectively, compared to 2.0 MPa for sebacic acid. Glass transition temperatures were also higher for HMDA (16.0 °C) and BHMT (12.4 °C) compared with sebacic acid (−1.4 °C). Moreover, TGA showed that samples cured using sebacic acid reached the point of fastest mass loss at lower temperatures (385 °C) than thermosets cured using amine hardeners (450–470 °C), indicating their improved thermal stability. However, HMDA samples exhibited a significant mass loss of up to 40% due to evaporation during curing. This study shows the potential of amine cross-linkers for enhancing performance and underscores the need for further research into optimizing curing conditions and cross-linking chemistry.

## Introduction

The replacement of traditional polymeric materials with biobased, biodegradable, and recyclable alternatives is becoming increasingly necessary due to environmental pollution and the anticipated depletion of fossil resources. Many everyday products require flexible materials with high bendability. Examples include clothing, seals, cushioning, packaging, tubing, adhesives, and coatings. Traditionally, silicones, synthetic rubbers, polyurethanes, conventional thermoplastics, and other types of fossil-sourced polymers have been used in these applications.

In the search for biobased solutions for flexible polymers, plant oils stand out among the classes of natural materials due to their unique molecular structure.^1^ Unsaturated plant oils such as linseed oil or soybean oil contain unsaturated fatty acids, which endow the oil with reactivity for polymerization and cross-linking.^1,2^ Autoxidative cross-linking of the long-chain fatty acids results in a loose network with elastomeric characteristics. The resulting material can be seen as a flexible biobased thermoset and has been used since ancient times for applications such as paints or wood coatings.^3^

Cross-linking of plant oil by autoxidation is a slow process, but the reactivity (and thus cross-linking rate) of unsaturated plant oils can be greatly improved by their conversion into epoxidized plant oils (EPOs). The most frequently reported method for the preparation of EPOs is the Prilezhaev reaction, which uses in situ formed peracetic acid or performic acid in the presence of a catalyst such as sulfuric acid.^4,5^ EPOs are typically derived from plant oils with high amounts of unsaturated fatty acids, specifically soybean oil or linseed oil.^6^ Because of their global industrial application as plasticizers, EPOs are produced in large quantities and thus represent a cheap and abundant renewable-based resource.^7^ Just as crude plant oils, EPOs are nontoxic and biodegradable and are therefore an interesting feedstock for the production of biobased materials.

A growing body of research has shown that EPOs can be converted into fully bioderived thermosets with flexible properties.^8−10^ In this context, many studies have focused on the use of aliphatic carboxylic acids because of their biobased character and high abundance. Some examples include adipic acid,^11,12^ sebacic acid,^8,12^ dodecanedioic acid,^12^ itaconic acid,^13^ citric acid,^14−16^ malic acid,^14^ tartaric acid,^14,15^ dimerized fatty acids,^17^ and various dicarboxylic acids of varying chain length.^18,19^

However, thermosets prepared from the reaction of EPOs with aliphatic carboxylic acids possess a limited mechanical performance. The tensile strength of the final materials is usually below 10 MPa, but in many cases, it does not exceed 2 MPa. Successful flexible materials typically balance bendability with strength to provide them with durability for long-term use. Leather is a good historical example: despite its flexibility, bovine leather has a tear strength of 15–50 MPa,^20,21^ while synthetic polyurethane-based leather alternatives typically achieve 10–20 MPa. Improvements can be gained by engineering composites and utilizing the reinforcement of fibers.^22,23^ A parallel approach is the improvement of the polymer matrix, which demands more studies on curing agents and cross-linking chemistry.

In contrast to well-studied aliphatic carboxylic acids, polyfunctional aliphatic amines have received relatively little attention as cross-linkers of EPOs, and their cross-linking chemistry is not well understood. To the best of our knowledge, there are only two sources describing the use of these hardener types, the first using decamethylene diamine^24^ and the second using hexamethylene diamine (HMDA).^25^ Both articles have described the cross-linking of epoxidized soybean oil (ESO). By contrast, epoxidized linseed oil (ELO) has a higher epoxy content and achieves a higher cross-link density, resulting in higher glass transition temperatures and mechanical properties than ESO.^26^ Despite the expected improved properties, the cross-linking of ELO with aliphatic amines has not yet been studied and described.

**Figure 1:**
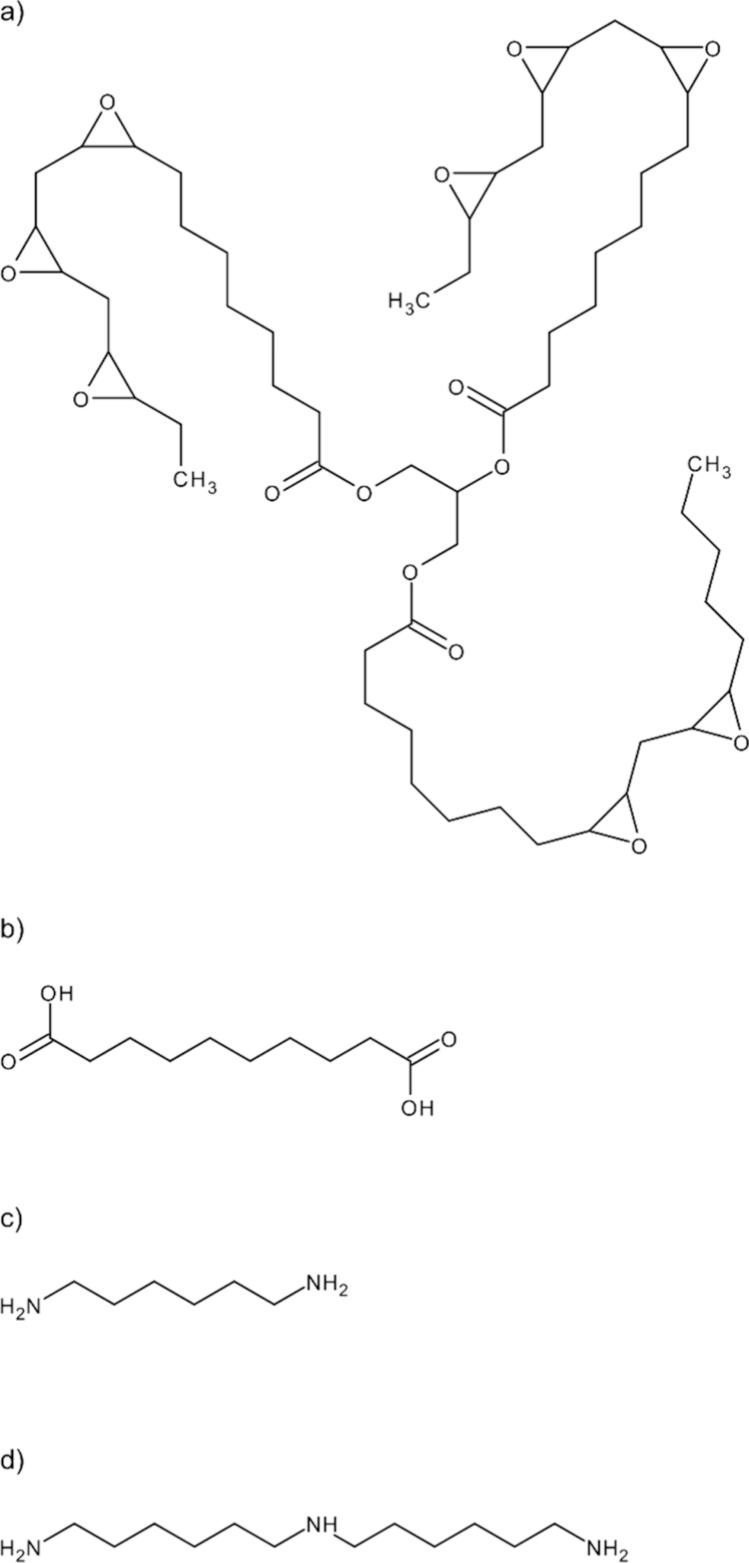
Chemicals used within this study: (a) exemplary structure of epoxidized linseed oil (ELO); (b) sebacic acid; (c) hexamethylene diamine (HMDA); (d) bis(hexamethylene)triamine (BHMT).

In this work, epoxidized linseed oil was cured using two types of amine cross-linkers: hexamethylene diamine (HMDA) and bis(hexamethylene)triamine (BHMT) (Figure 1). HMDA is a commodity chemical used by the nylon industry on the order of 2 million tons per year globally. While conventionally produced from fossil feedstocks, it is possible to produce HMDA from biomass, such as HMF, by various methods, including enzymatic fermentation.^27,28^ Recently, industry players have expressed interest in large-scale production of biobased HMDA.^29^

BHMT is a trifunctional amine hardener and structurally a dimer of HMDA and has not yet been described in the literature as a cross-linker of epoxidized plant oil. For comparison reasons, ELO was also cross-linked by sebacic acid, a biobased aliphatic dicarboxylic acid, similar to the work reported by Schwaiger et al.^8^ The same conditions were used in the curing of all three hardeners to allow for a comparative study on the hardeners’ performance.

## Experimental Section

### Materials

Epoxidized linseed oil was obtained from HOBUM Oleochemicals GmbH (Germany). An epoxy oxygen content of 8.52 wt % was indicated by the supplier. Hexamethylene diamine (98%, CAS 124-09-4), bis(hexamethylene)triamine (>94%, CAS 143-23-7), and sebacic acid (≥95%, CAS 111-20-6) were all obtained from Sigma-Aldrich Handels GmbH (Austria).

### Preparation of Thermosets

Thermosets were produced by mixing epoxidized linseed oil with varying amounts of either sebacic acid (SEBA), bis(hexamethylene)triamine (BHMT), or hexamethylene diamine (HMDA) as hardeners. For the preparation of thermosets based on sebacic acid, ELO and sebacic acid were combined in a beaker and heated and stirred at 500 rpm on a hot plate until sebacic acid fully melted (∼140 °C). Thermosets based on HMDA and BHMT were produced by first melting the amine at 50–60 °C in a water bath. ELO was preheated to 100 °C, and then warm amine hardener was added to it. The components were mixed for 1 min at around 500 rpm to mix the material. About 5 g of the hot liquid ELO-hardener mixtures was quickly transferred into C-type dog bone shaped silicon mold (total length = 115 mm, width at center = 6 mm, depth = 2 mm). Samples were cured for 24 h at 140 °C. This temperature was chosen based on previous studies on sebacic acid,^8^ and the results of a preliminary curing study (Figure S1). To decrease adhesion between sample and silicon, a Teflon spray was applied to the molds of samples cured using HMDA and BHMT.

### Fourier Transform Infrared (FT-IR) Spectroscopy

Fourier transform infrared (FT-IR) spectroscopy was conducted to compare the chemical properties of samples cured with varying amounts of hardener. To ensure an accurate comparison of bulk properties, the top layer of each sample was removed by using a razor blade. Both the bulk properties and the surfaces of the samples were analyzed. FT-IR measurements were performed by using a PerkinElmer Frontier instrument with ATR sampling. For each spectrum, 16 scans were recorded in the range of 500–4000 cm^–1^. Each sample was measured in duplicate. The spectral data were processed using Spectragryph 1.2. This involved baseline fitting and averaging of duplicate measurements.

### Differential Scanning Calorimetry (DSC)

Differential scanning calorimetry (DSC) measurements of cured resins were performed on a Polyma 214 instrument (Netzsch GmbH). Samples, each weighing 14.0 ± 1.5 mg, were cut from the bulk and transferred to aluminum crucibles. Samples were cooled to −60 °C, and heated to 100 °C at 10 K/min under a nitrogen atmosphere. This cycle was repeated twice, and the glass transition temperature (*T*_g_) was determined from the second heating curve using NETZSCH Proteus Analysis (version 8.0.3).

### Dynamic Mechanical Analysis (DMA)

DMA was measured in tensile mode with the Dynamic Mechanical Analyzer 242 C (Netzsch GmbH), at an oscillation frequency of 1 s^–1^, from 65 to 90 °C at a heating rate of 10 K/min. Samples were cut to appropriate dimensions from the dog-bone samples using a razor blade. Dimensions of the testing area were approximately 10 mm length, 5 mm width, and 2–3 mm thickness. Exact dimensions were determined for each sample with a cantilever.

### Tensile Tests

Tensile tests were conducted in accordance with ASTM D412. Dog bone-shaped samples, prepared as described in the Supporting Information, were tested until fracture using the Zwick-Roell 2020 (Zwick GmbH & Co. KG, Ulm) universal testing machine with a preload of 0.5 N and a crosshead speed of ∼500 mm/min. The sample thickness was determined by calculating the mean of three measurements.

### Thermogravimetric Analysis (TGA)

TGA of synthesized resins was performed using a TG 209 F1 Libra instrument (Netzsch). Measurements were conducted under nitrogen flow from 30 to 600 °C at a heating rate of 10 K min^–1^ and using a sample mass of 15 ± 1 mg.

### Gel Content Analysis

To determine the gel content (GC), samples measuring 10 mm × 10 mm were cut from the individual specimens. The initial oven-dry mass (*m*_i_) was determined after drying in a vacuum oven at 50 °C. Each of the dry samples was immersed in 5 mL of acetone or toluene for 48 h. Thereafter, samples were dried at 70 °C in an oven until their mass remained constant, and their final mass (*m*_f_) was determined. The GC (%) was calculated using the following equation:
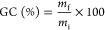


### Water Uptake

Water uptake was carried out in alignment with EN ISO 62. Briefly, samples of 1 × 1 cm^2^ were conditioned at 50 °C until their mass was constant and then immersed in distilled water. The mass was determined after 24, 48, and 72 h of immersion.

## Results and Discussion

### Fourier Transform Infrared Spectroscopy

**Figure 2:**
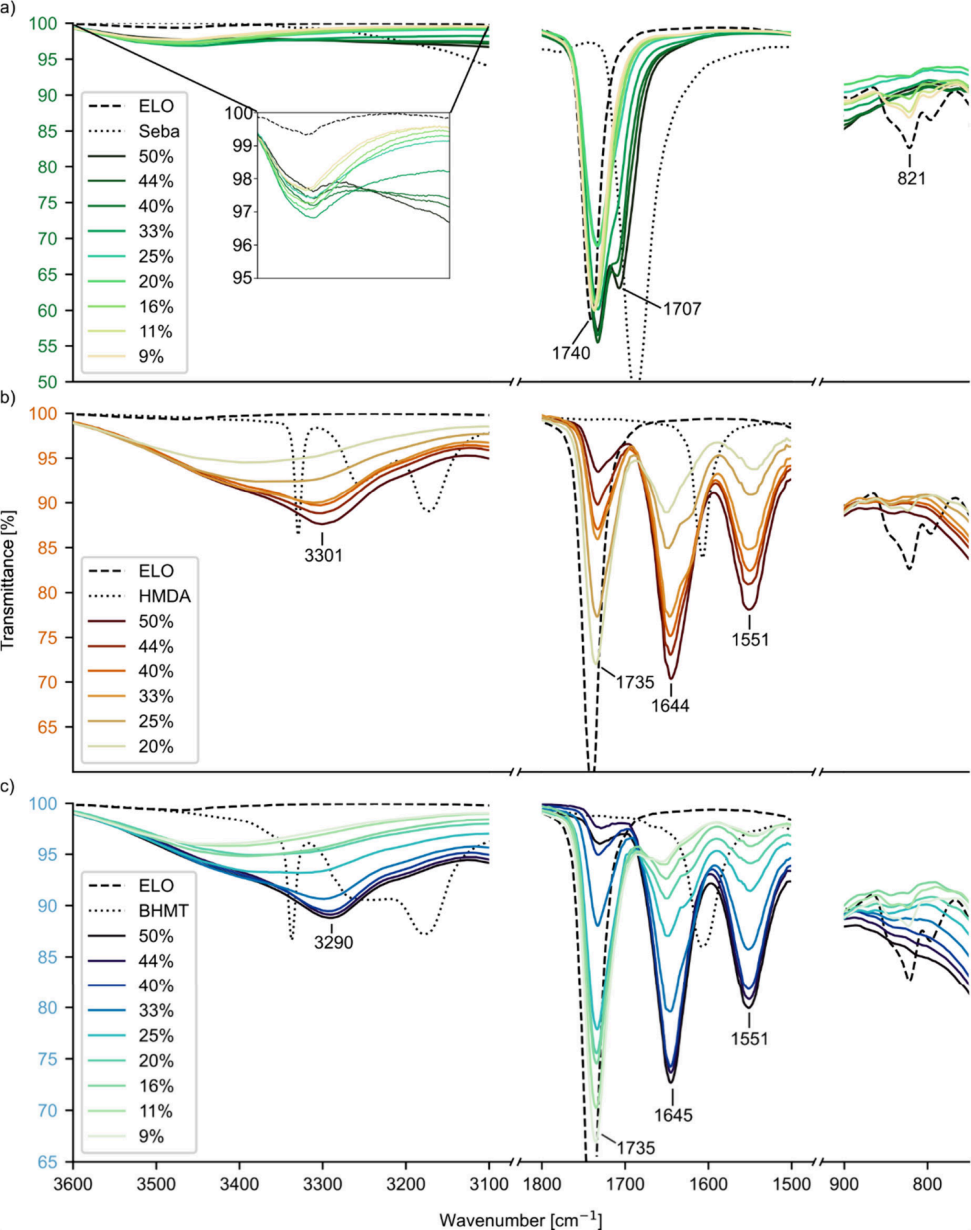
FT-IR spectroscopy: relevant bands of epoxidized linseed oil cured with varying concentrations of sebacic acid (a), hexamethylene diamine (b), and bis(hexamethylene)triamine (c).

**Scheme 1:**
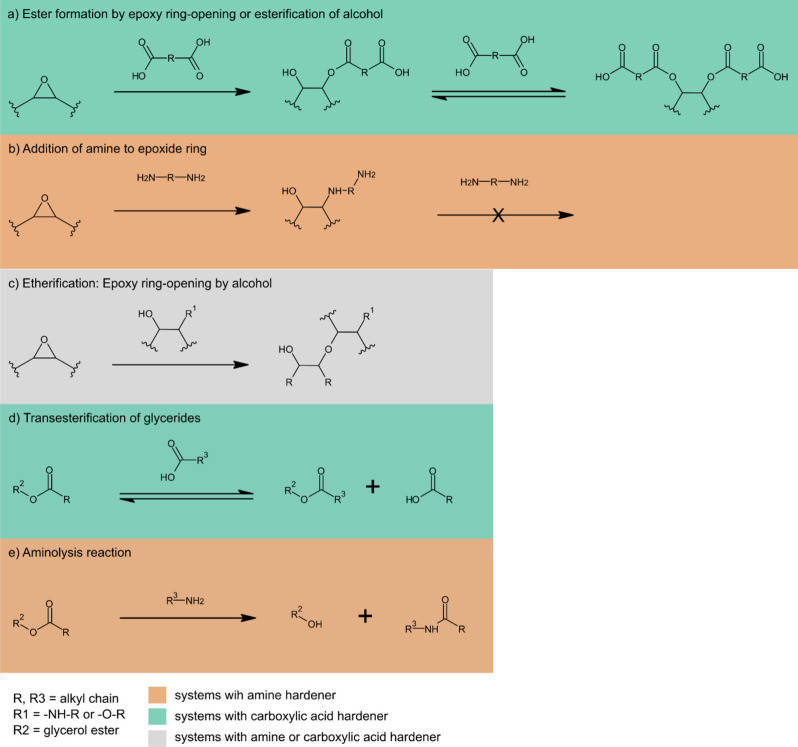
Possible Reactions Occurring during the Cross-Linking of the Thermosets Prepared in This Study: (a) Ester Formation, (b) Amine Addition, (c) Etherification, (d) Transesterification, (e) Aminolysis

Figure 2 presents the results of FT-IR measurements of the inner surfaces of ELO samples cured with either sebacic acid (a), HMDA (b), or BHMT (c). The broad O–H stretching band was visible for all thermoset samples, and the intensity of this band increased with increasing hardener load. This is related to the opening of the epoxide rings during curing with concomitant generation of free hydroxyl groups (Scheme 1a–c).^8^ This effect was well observed for HMDA and BHMT, yet it was much less pronounced for sebacic acid. One explanation for this can be the esterification of the generated OH groups with COOH groups of sebacic acid, as described in the literature (Scheme 1a).^8,30^ In the cases of HMDA and BHMT, generated OH groups are not consumed, as they are in the case of sebacic acid, because alcohols and amines are unable to react with each other under the present conditions. Moreover, vibrations resulting from NH groups can contribute to the prominence of this area, so that samples cured with higher amount of amine hardeners show a peak at around 3300 cm^–1^.^25,31^ This peak is related to the N–H stretching band of amine or amide^25,31^ and becomes clearly apparent for BHMT and HMDA contents ≥25%. The progressing reaction is best understood by looking at the epoxide band. Uncured epoxidized linseed oil (ELO) shows a strong epoxide band at 821 cm^–1^ which decreases with increasing concentration of cross-linker and finally vanishes in cases of higher cross-linker amounts. This is true for all three cross-linker types and gives proof for the–eventually complete–consumption of epoxide groups during the curing process. In the case of HMDA and BHMT, the disappearance of the epoxide band coincides with the appearance of the amine band. Although it is not possible to distinguish the secondary amine groups from unreacted primary amine groups in the FTIR spectrum, a possible explanation can be the presence of excess amine after consumption of epoxy moieties in ELO, at elevated concentrations of BHMT and HMDA. Below, we will see that this specific ratio of reacting groups also leads to the highest mechanical and *T*_g_ properties.

More insight into the curing reaction can be obtained from the carbonyl area between 1600 and 1800 cm^–1^. Pure ELO shows a strong C=O stretching band of the triglyceride esters at 1740 cm^–1^. The reaction of sebacic acid with epoxide groups also causes ester formation (Scheme 1a). At hardener concentrations of 33% and above a shoulder at 1707 cm^–1^ arises due to the presence of excess dicarboxylic acid, as also described in literature^8,32^ Similar to the case of HMDA and BHMT, the concentration where the epoxide band disappeared and the band from nonreacted COOH groups was still absent (25% of sebacic acid) marked the point of optimal consumption of functional groups.^8^

In the spectra of samples cured with HMDA and BHMT, the ester peak at 1735 cm^–1^ is also present, but contrary to sebacic acid, its intensity significantly decreases upon the increase of the hardener. Simultaneously, the intensity of the amide carbonyl band at 1644/1645 cm^–1^ increases in intensity. The same observation holds for the peak at 1551 cm^–1^, which is likely due to N–H bending. Both peaks indicate the formation of amides in the reaction.^25^ As the only possible reaction partner for amide formation is the fatty acids in the oil, the reaction must proceed by aminolysis of the triglyceride esters (Scheme 1e).^24,25^ The weak remaining ester carbonyl band at higher hardener concentrations suggests that at this stage, most of the fatty acids in the material had been converted into amides. This mechanism also includes the generation of free glycerol hydroxyl groups, which contribute to the increasing intensity of the OH band in samples cured using BHMT and HMDA (Scheme 1e).

### Sample Curing and Mass Loss

In a preliminary curing study (Figure S1), an exothermic curing peak in DSC was found starting at temperatures in the range of 130–140 °C, depending on the hardener. A previous study using sebacic acid for epoxidized linseed oil had used a curing protocol of 24 h at a temperature of 140 °C.^8^ This protocol was adopted herein for all samples, as it should allow full conversion of all variants at relatively benign temperatures.

**Figure 3:**
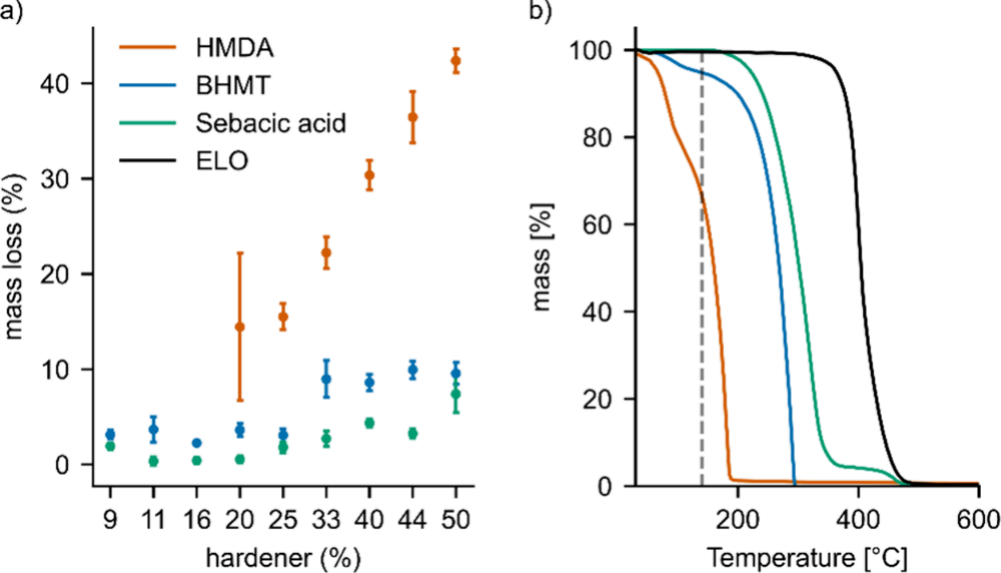
(a) Mass loss of samples during preparation. (b) Thermogravimetric analysis of pure components. The dotted line represents a curing temperature of 140 °C.

During preparation of the test specimens, a decrease in sample thickness became apparent. Sample mass was thus measured, showing that some samples lost weight during curing. Figure 3a shows the respective decreases in sample mass as a function of hardener content in the samples. Thermogravimetry performed on pure epoxidized linseed oil and pure hardeners showed that at the curing temperature of 140 °C, both sebacic acid and ELO had a negligible mass loss <1%, while BHMT and HMDA showed significant mass loss of 5% and 33%, respectively (Figure 3b). The lower mass loss observed in Figure 3b compared with Figure 3a can be attributed to the shorter exposure time to the temperature in the TGA, as opposed to the prolonged exposure during oven curing. The results suggest that the mass loss is caused by the evaporation of hardeners. The much lower boiling point of 204.6 °C for HMDA versus 294.4 °C for sebacic acid also aligns with this interpretation. In general, higher hardener content also led to greater mass loss. For sebacic acid and BHMT, the mass loss of all samples was below 10%. By contrast, mass loss for HMDA exceeded 40% for the highest tested hardener content. Although plant oil epoxy cured with HMDA has been described before, this observation has not yet been reported.^25^ The choice of BHMT in this study was partly due to the necessity to use an amine cross-linker being less volatile than HMDA.

Samples containing less than 20% of HMDA were not prepared in this study, as pre-experiments showed incomplete curing, which can be explained by an insufficient amount of hardener left after evaporation. The high mass loss, especially for HMDA samples, makes it hard to determine the exact amount of hardener in the final material. Therefore, throughout this study we refer to only the initial hardener content.

### Mechanical Properties

**Figure 4:**
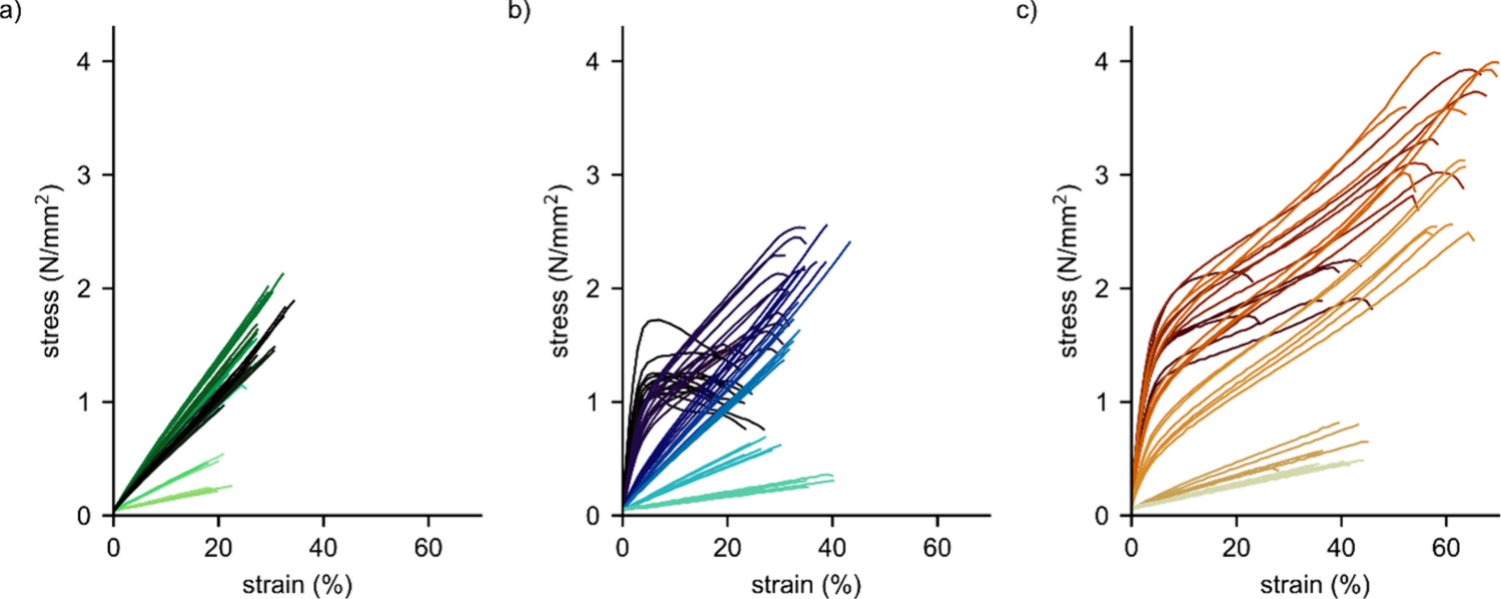
Stress–strain diagrams for ELO cured with sebacic acid (a), BHMT (b), and HMDA (c). Color intensity reflects the amount of hardener added.

To investigate the mechanical properties of cured thermosets, samples were tested in tensile mode on a universal testing machine. Figure 4 displays data from all tensile tests performed in this study in plots with equal dimensions to emphasize differences in mechanical properties among the three hardeners. Color intensity reflects the amount of hardener added. Several trends can be derived: The highest achieved tensile strength and elastic modulus increase in the order of sebacic acid < BHMT < HMDA. Similarly, elongation at break is highest for HMDA samples and lowest for sebacic acid samples. In each group of hardeners, certain hardener concentrations are distinguished by specific mechanical properties. The highest strength was not achieved by the highest hardener concentrations but by an intermediate concentration, which we define as optimum hardener concentration.

**Figure 5:**
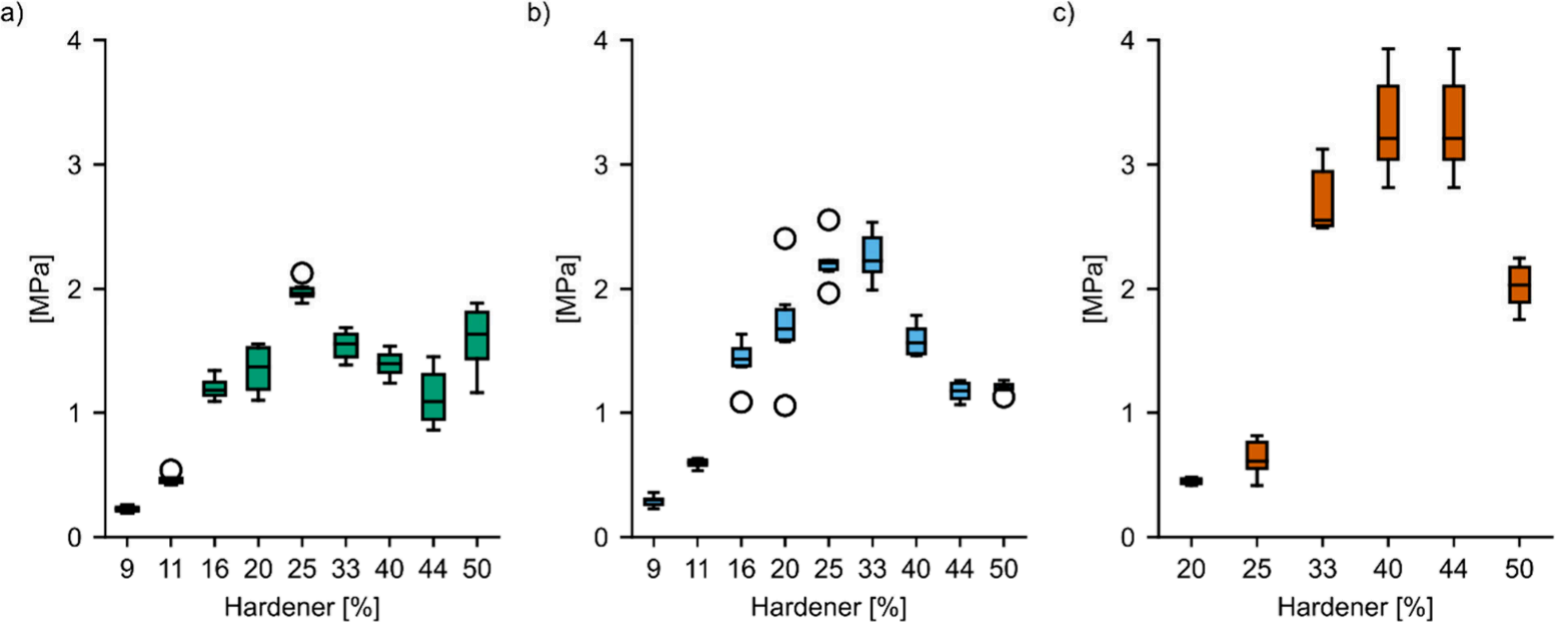
Ultimate tensile strength of ELO cured with sebacic acid (a), BHMT (b), and HMDA (c).

Figure 5 provides deeper insight into this effect, showing how ultimate tensile strength peaks at concentrations of 25% sebacic acid (*R* = 0.62), 33% BHMT (*R* = 1.29), and 40% HMDA (*R* = 2.15), with *R* meaning the initial molar ratio of carboxylic acid or amine groups to epoxide moieties. Higher and lower levels of hardener led to a decreased strength. This is best explained by the fact that the highest cross-linking density is achieved at optimal ratios of reacting functional groups. For dicarboxylic acids, this is well documented in the literature, where values of an *R*-value equal or close to 0.70 have been reported as optimum.^8,12^ This is close to the value of 0.62 reported here. The fact that HMDA required a much higher theoretical *R* ratio than sebacic acid can be related to the evaporation of HMDA. The highest average tensile strengths in each hardener group were 3.7 MPa for 40% HMDA, 2.3 MPa for 33% BHMT, and 2.0 MPa for 25% sebacic acid. The strength of ELO cured with sebacic acid agrees well with the tensile properties reported in the literature (2–3 MPa).^8,18^

Intarabumrung et al.^25^ cured ESO with different amounts of HMDA and achieved a maximum of 2.44 MPa for the best-performing samples. The higher tensile strength reported herein for HMDA can be related to the fact that cross-linked ELO delivers better mechanical properties than ESO, which is well-documented.^12,18^

An obvious reason why HMDA may provide improved properties compared to those of sebacic acid is its shorter chain length. ELO and ESO have been cured with hardeners of various length with shorter chain lengths performing better in terms of mechanical properties.^12,18^ Ding et al.^18^ reported that adipic acid with a chain length of C6 reached 8.8 MPa and was thus well-above the other hardeners, while most longer-chain hardeners performed in the range of 1–4 MPa. The authors related this effect to chain straining, which could also play a role in samples cured with HMDA.

Due to the sample geometry, absolute values for elongation and Young’s modulus cannot be derived, but the results allow for a relative comparison between hardeners (Figures S2 and S3). While sebacic acid and BHMT had similar values for the elongation at break, the toughest HMDA samples achieved roughly twice the strain of the toughest samples cured with the other two hardeners. The strain at break of the HMDA samples with optimum hardener concentration was at least 50%, emphasizing the high ability to deform without rupture.

Samples cured with different hardeners showed clear differences in their E-moduli (Figure S3, logarithmic scale). The best-performing samples, cured with BHMT, had about a 7-fold increase in the E-modulus compared to those cured with sebacic acid. A similar increase was also seen in samples cured with HMDA compared to those cured with BHMT. Schwaiger et al.^8^ have reported Young’s moduli of 4–8 MPa for samples cured with sebacic acid, which may help to relate the values herein to absolute ones.

### Thermal Analysis of Cured Thermoset Samples

**Figure 6:**
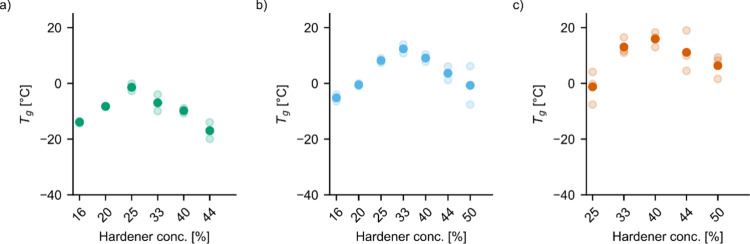
Glass transition temperature values determined from DSC thermograms of epoxy thermosets cured by using sebacic acid (a), BHMT (b), or HMDA (c). Bold color dots represent the mean calculated from at least two measurements, represented by faded dots.

DSC measurements were conducted on cured samples to derive the glass transition temperature (*T*_g_) of samples cured with varying concentrations of the three hardeners (Figure 6). Samples having <16% BHMT or sebacic acid or <20% HMDA exhibited ambiguous glass transitions or more than one glass transition, and their results are not displayed. Similar to the results of mechanical testing, glass transition temperatures peaked in each group for a specific hardener concentration and decreased at both higher and lower concentrations. *T*_g_ peaked at concentrations of 25% sebacic acid, 33% BHMT, and 40% HMDA. Thus, the maximum *T*_g_ in each group coincides with the ratio providing the best mechanical performance: a stronger molecular network both improves strength and decreases the mobility of molecular segments, leading to higher *T*_g_. Also, between groups, the trend matched the observations for mechanical properties, with maxima of *T*_g_ increasing from −1.4 °C for sebacic acid to 12.4 °C for BHMT and 16.0 °C for HMDA. The lower values of BHMT can be explained by its greater chain length compared to HMDA. Although BHMT does have three amine functionalities, the central amine group is a secondary one, which is less accessible and thus less reactive than the terminal primary amine groups. This reduction in the overall cross-link density is reflected by the lower maximum *T*_g_ value compared to samples cured by HMDA.

There are no reports on testing the *T*_g_ of epoxidized plant oil cured with amines. However, several studies have used DSC to determine the *T*_g_ of EPO cured with dicarboxylic acids. Ding et al.^18^ cured ELO at *R* = 0.70 with different dicarboxylic acid hardeners with chain lengths varying from C_6_ to C_36_, and obtained *T*_g_ values of −15.1 to 7.0 °C. The authors measured a *T*_g_ of 0.4 °C for sebacic acid, which is very similar to the value obtained in this study at *R* = 0.62. For adipic acid, with the shortest carbon chain length in that study, the authors measured the highest *T*_g_ of 7.0 °C. Just as adipic acid, HMDA has a carbon chain length of C_6_. The fact that HMDA in this study achieved 16.0 °C suggests that with the amine-based variant higher glass transition temperatures are possible than with the carboxylic acid counterparts of the same chain length, potentially related to other reactions, such as amide formation.

**Figure 7:**
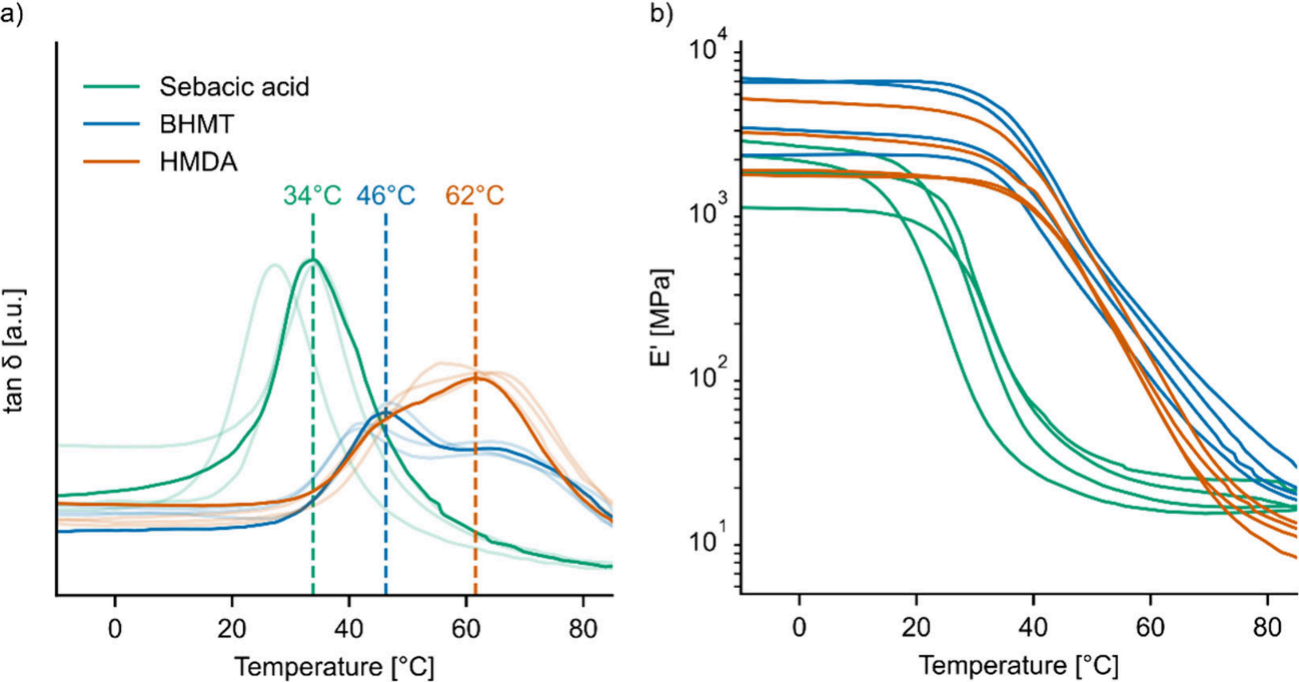
Results of DMA measurements for ELO cured at the best-performing ratio of hardeners (25% sebacic acid, 33% BHMT, and 40% HMDA). The tan δ evolution (a) and the storage modulus *E*′ (b) are shown for four samples per variant.

Dynamic mechanical analysis (DMA) was performed in tensile mode on selected thermosets, which had shown the highest glass transition values for each variant, specifically 25% sebacic acid, 33% BHMT, and 40% HMDA. Results for tan δ, which is the ratio of storage modulus and loss modulus, are presented in Figure 7. The maximum value of tan δ is assigned to the α relaxation (*T*_α_) phenomenon which is related to the cooperative motions of polymeric chains, associated with the *T*_g_, but gives higher values than the *T*_g_ measured by a change in *c*_p_ in DSC. By comparing the thermosets developed with sebacic acid, BHMT, or HMDA hardeners, we can observe in Figure 7 that the networks show a more Gaussian shape of the tan δ peak for the polyester resin developed with ELO/sebacic acid. This is an indication of a homogeneous distribution of the relaxation times in the polymer motions. In contrast, a more complex tan δ shape can be seen in the networks developed with aliphatic BHMT and HMDA tri- respectively diamines. The mean of tan δ_max_ followed the same order as in the DSC measurements: sebacic acid (32 °C) < BHMT (44 °C) < HMDA (63 °C). While ELO cured with sebacic acid only showed a single peak, two overlapping peaks were observed for ELO cured with HMDA or BHMT. For both amine hardeners, the first peak appeared at around 46 °C, and a second one at around 62 °C. For the networks based on BHMT the first peak is more prominent, while the second one is dominant in ELO cured with HMDA. The appearance of multiple peaks in tan δ has in the literature been interpreted as an indication of the presence of more than one cross-linked network with different rigidity.^25,33^ The fact that ELO-sebacic acid resin has a higher tan δ peak amplitude is showing an increased energy dissipation by internal friction due to more free volume and increased chains motions. At the opposite are the systems with ELO-BHMT and ELO-HMDA amines where the first α relaxation peaks (∼46 °C) have a similar amplitude, much lower than that of ELO- sebacic acid. This lower peak height can be explained by more constrained polymer chains motions, perhaps due to hydrogen bonding between N–H of amide and carbonyl oxygen, as described for polyamides.^25^ The storage modulus of the samples covered a rather wide range from 1 to 7 GPa. The higher variability can also be explained by the fact of the softness of the samples, which were easily damaged during clamping in the DMA. As a general trend, it appears the *E*′ at temperatures < *T*_g_ (b) was increased in the amine samples compared to sebacic acid samples, which matches the observation of higher stiffness of the samples in tensile testing. Moreover, this drop in *E*′ occurs at much higher temperatures than in the sebacic acid samples, meaning that this stiffness was preserved to higher temperatures. To summarize, the use of amine hardeners seemed to form a more rigid network with higher glass transition temperatures and storage moduli compared to those of the carboxylic acid hardener.

### Thermogravimetric Analysis

**Figure 8:**
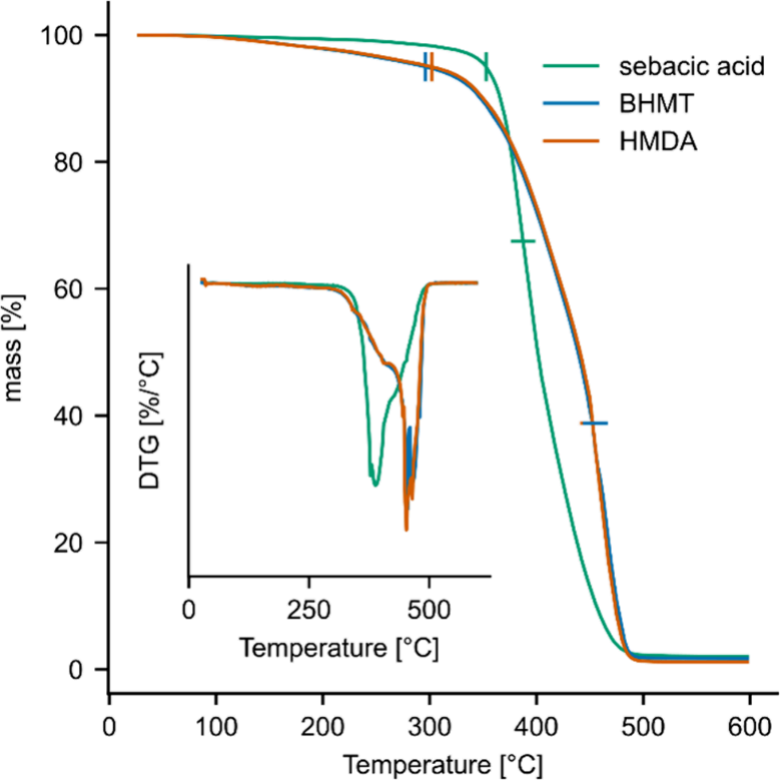
Thermogravimetric analysis of samples cured with best-performing concentrations of the three different hardeners, 25% sebacic acid, 33% BHMT, and 40% HMDA. Vertical lines indicate the temperature at 5% mass loss, and horizontal lines indicate *T*_max_, the temperature of most rapid mass loss.

Thermogravimetric analysis (TGA) was conducted under nitrogen to investigate the thermal degradation behavior of the samples cured with the optimum hardener concentration of 25% sebacic acid, 33% BHMT, or 40% HMDA (Figure 8). All samples were fully degraded at a temperature below 500 °C and residual masses were between 1–2%. The degradation behaviors of samples cured with BHMT and HMDA were similar, whereas samples cured with sebacic acid showed different degradation curves. Samples cured with amine hardeners were characterized by an earlier decrease in mass, with mass loss already starting at values below 150 °C. This early mass loss was absent in samples cured using sebacic acid and could be either due to the degradation of weak bonds, such as ammonium ion pairs, or due to the evaporation of remaining unreacted hardener from the sample. A mass loss of 5% was reached at temperatures around 280–300 °C, similar to what has been reported in the literature for ESO cured with HMDA.^25^ Conversely, samples cured with sebacic acid reached a 5% decrease in mass only at a temperature above 350 °C, similar to the value of 356.1 °C reported by Ding et al.^18^ Thereafter, the degradation curve of the sebacic acid samples is characterized by a rapid decrease in mass. The inset in Figure 8 plots the first derivative of the mass loss (DTG), showing that in samples cured with sebacic acid the point of fastest mass loss (*T*_max_) was reached at around 385 °C. Conversely, the amine hardeners reached their *T*_max_ only at 450–470 °C, which is close to the reported literature value.^25^ The DTG diagram further shows that the cleavage of the amine-cured thermosets follows a two-stage thermal decomposition. The first stage, visible in the DTG inset as a small shoulder at around 400 °C, has been related to the degradation of ester groups.^24,25^ The second stage, here characterized by a sharp peak at around 450–470 °C, has been related to amide bond degradation.^24,25^ Sebacic acid-cured samples conversely had a sharp peak at 390 °C and a smaller shoulder at about 430 °C. The first degradation has been attributed to ester linkages of ELO, and the second due to ester linkage of acid and epoxy groups.^18^ To summarize, the results suggest that the amide network generally provides the amine-cured samples with improved thermal stability, slowing down the degradation, while minor mass loss starts earlier in these samples.

### Analysis of Gel Content (GC %)

The determination of the gel content (GC %) of samples after curing was carried out to assess the extent of cross-linking in the polymer network (Table 1). Samples were immersed in acetone or toluene for a duration of 24 h, and the GC (%) was determined from their oven-dry mass before and after storage in the solvent. All samples exhibited slightly higher stability in toluene than in acetone, suggesting that acetone could better dissolve the unreacted monomers or oligomers formed in the cross-linking. All tested samples showed a high GC (%) above 85%, indicating the formation of a cross-linked network. The gel content was higher in the group cured with BHMT, followed by HMDA and sebacic acid. This aligns with the superior thermomechanical properties of amine-cured samples observed in tensile testing and DMA measurements. Moreover, in each group, the cross-linking density was highest for samples showing the highest *T*_g_ and mechanical properties. For samples cured using amines, this was the case for hardener contents of 33% and 40%, while for sebacic acid, the highest GC (%) was achieved with 25% hardener content. This emphasizes the influence of appropriate epoxy-hardener ratios for achieving highly cross-linked networks with optimal thermomechanical performance.

**Table 1:** Values of the Gel Content of ELO Cured with the Three Studied Hardeners at Best-Performing Hardener Amounts of 25%, 33%, and 40%^a^

hardener type	hardener concentration (wt %)	GC (%), acetone	GC (%), toluene
sebacic acid	25	95.6	97.9
	33	91.5	96.9
	40	86.9	95.3
HMDA	25	88.1	95.9
	33	96.6	99.1
	40	97.3	98.5
BHMT	25	96.7	98.7
	33	98.1	99.5
	40	99.7	99.8

a The averages of a 2-fold determination in either acetone or toluene are presented.

### Investigation of Bulk and Surface Properties of Samples

**Figure 9:**
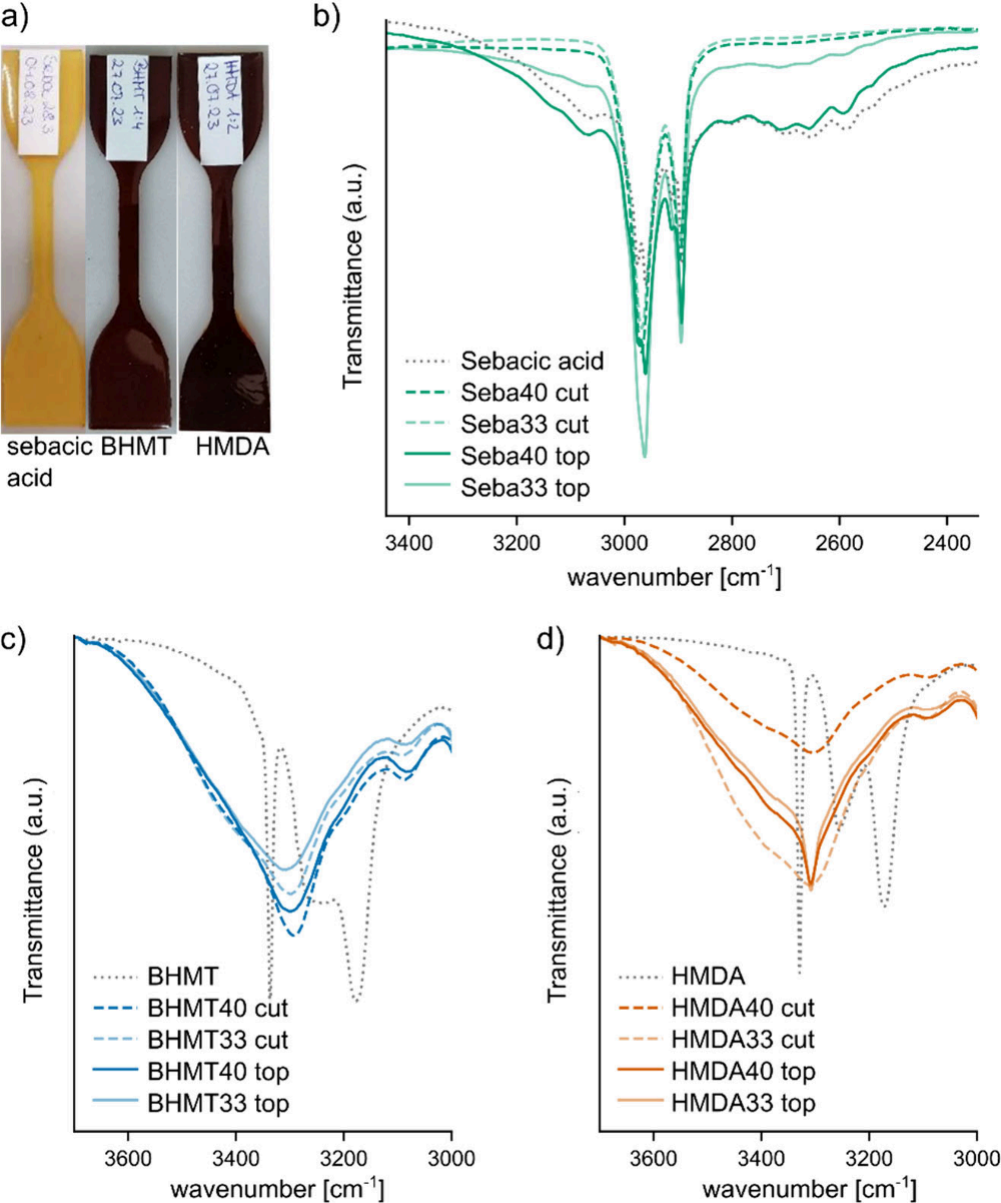
Images of tensile samples of ELO cured with sebacic acid, BHMT, or HMDA (a). FTIR of cut inner surfaces and top surfaces of ELO cured with sebacic acid (b), BHMT (c), or HMDA (d).

Images of the thermosets cured with the three types of hardeners are shown in Figure 9a. A strong coloration of samples prepared with amine curing agents was evident. Cutting of the samples showed that the inner surfaces were yellow translucent, as is typical for cured ELO and that the brown coloration was present only at the surface. This led to the question of differences between the surface and bulk and whether the distribution of the chemicals was homogeneous throughout the cross-section of samples. Raman microscopy was conducted on several spots on the cross-section to better understand potential spatial inhomogeneity (Figure S4). Raman spectra of ELO cured with sebacid acid were homogeneous along the cross-section. In samples cured by using HMDA and BHMT, areas closer to the surface caused increased signal noise. This effect was present in areas close to the top and the bottom of samples but absent in inner sections. Exact determination of the chemical differences between the outer and inner areas has not been attempted. However, the presence of noise in the Raman spectra is related to fluorescence, providing proof of the presence of chromophore groups close to the surface. A possible explanation for colored compounds can be reactions of amines with oxygen from the air, and a higher local basicity catalyzing chromophore-generating condensation reactions.^34,35^

Additionally, FT-IR was conducted on outer surfaces (“top”) and cut surfaces (“cut”) of samples cured with hardener contents of 33% and 40% to understand chemical differences between the surface and the bulk of samples. Full FT-IR spectra are shown in Figure S5. For samples cured using sebacic acid, a strong carbonyl peak at ∼1700 cm^–1^ related to nonreacted acid is present for top samples. Cut samples show only the carbonyl peak of esters at ∼1740 cm^–1^, while the peak related to carboxylic acid is absent. Moreover, in the range of 2400–3400 cm^–1^, a band related to the O–H stretching of carboxylic acid is present (Figure 9b). Both observations provide proof of the presence of unreacted sebacic acid on the samples’ surface. Furthermore, bands related to carboxylic acid were more prominent at a concentration of 40% than at 33%, pointing to the accumulation of unreacted carboxylic acid at higher hardener concentrations. Figure 9b and Figure 9c show the area around 3000–3600 cm^–1^ of cut and top samples cured with BHMT and HMDA. While peak shapes in this region are similar for cut and top samples prepared from BHMT, top samples cured with HMDA show sharp distinct peaks at 3300 cm^–1^, which are absent in cut samples. This, once again, is proof of the accumulation of unreacted amine groups at the samples’ surface-near regions.

### Interaction with Water

**Figure 10:**
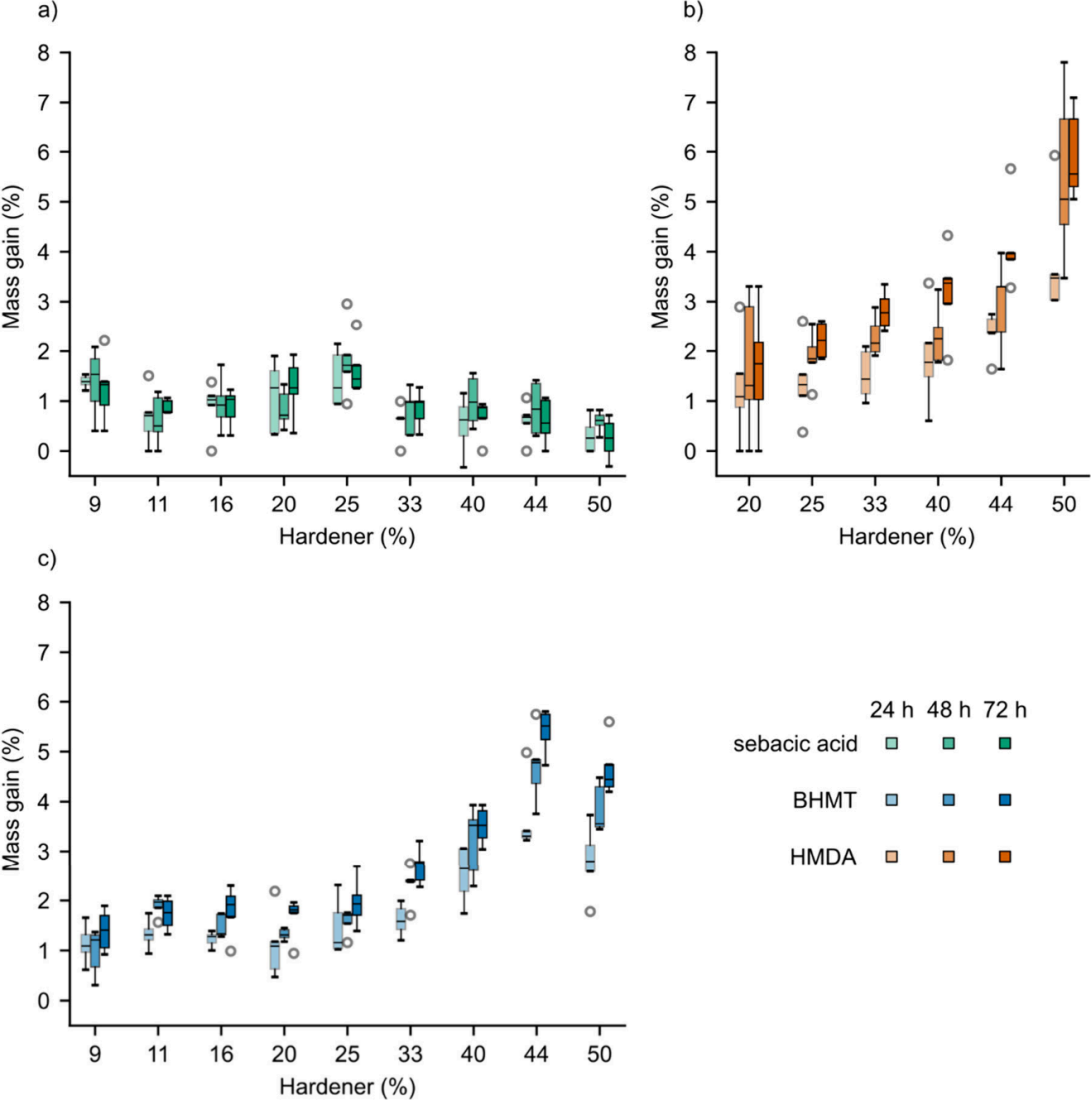
Mass gain for samples cured using sebacic acid (a), HMDA (b), or BHMT (c) subjected to water treatment.

To assess the water-absorption, samples were immersed in water for 24, 48, and 72 h, with the durations being represented by light, intermediate, and strong color intensities in Figure 10. Samples cured with sebacic acid showed a mass gain of <2%, and the mass gain was independent of the duration of treatment. In samples cured with HMDA and BHMT, mass gain increased with higher concentrations of hardener and peaked at the highest concentrations of 44% and 50%. Moreover, longer treatment time contributed to absorption. The higher water absorption of BHMT and HMDA samples can be related to the higher polarity of HMDA and BHMT compared to sebacic acid, increasing the overall polarity of the produced materials. At higher concentrations, free unreacted end-groups may be present, which at neutral pH would exist in their protonated form, which may also increase polarity and contribute to the interaction with water.

## Conclusion and Outlook

This study demonstrates the preparation of biobased thermosets with flexible properties by cross-linking of epoxidized linseed oil (ELO) with either hexamethylene diamine (HMDA), bis(hexamethylene)triamine (BHMT), or sebacic acid. The mechanical and thermal analyses showed that amine cross-linkers, particularly HMDA, significantly enhance the tensile strength and glass transition temperature of the resulting thermosets compared to sebacic acid. However, the high evaporation rate of HMDA during curing poses a challenge that must be addressed. Future research should focus on optimizing the curing process to minimize mass loss and exploring alternative amine cross-linkers with lower volatility. Additionally, investigating the long-term durability, environmental impact, and potential toxicity of these biobased thermosets will be a crucial prerequisite to their practical application. We hope that the results of this study contribute to a better acceptance and wider application of biobased thermoset materials.

This study demonstrates the preparation of biobased thermosets with flexible properties by cross-linking epoxidized linseed oil (ELO) with either hexamethylene diamine (HMDA), bis(hexamethylene)triamine (BHMT), or sebacic acid. The mechanical and thermal analyses showed that amine cross-linkers, particularly HMDA, significantly enhance the tensile strength and glass transition temperature of the resulting thermosets compared to sebacic acid. However, the high evaporation rate of HMDA during curing poses a challenge that needs to be addressed. Additionally, HMDA and BHMT are corrosive aliphatic amines that may cause damage to the skin and eyes and irritate the respiratory tract, necessitating the use of personal protective equipment and well-ventilated workspaces. In contrast, carboxylic acids, such as sebacic acid, are less hazardous and not classified as corrosive, making them safer to handle. Future research should focus on optimizing the curing process to minimize mass loss and exploring alternative amine cross-linkers with lower volatility. Additionally, investigating the long-term durability, environmental impact, and potential toxicity of these biobased thermosets will be a crucial prerequisite to their practical application. We hope that the results of this study contribute to a better acceptance and wider application of biobased thermoset materials.

## Supporting Information Available

(1) Differential scanning calorimetry of ELO mixed with the 3 hardeners (Figure S1); (2) elastic modulus and normalized elongation at break (Figures S2 and S3); (3) Raman spectroscopy (Figure S4); (4) full FT-IR spectra (Figure S5) (PDF)

## References

[ref1] Montero de Espinosa L.; Meier M. A. R. Plant Oils: The Perfect Renewable Resource for Polymer Science?!. Eur. Polym. J. 2011, 47 (5), 837–852. 10.1016/j.eurpolymj.2010.11.020.

[ref2] Lligadas G.; Ronda J. C.; Galià M.; Cádiz V. Renewable Polymeric Materials from Vegetable Oils: A Perspective. Mater. Today 2013, 16 (9), 337–343. 10.1016/j.mattod.2013.08.016.

[ref3] Poth U. Drying Oils and Related Products. In Ullmann’s Encyclopedia of Industrial Chemistry; Wiley-VCH Verlag GmbH & Co. KGaA: Weinheim, Germany, 2001; pp 621–636 10.1002/14356007.a09_055.

[ref4] Wai P. T.; Jiang P. P.; Shen Y. R.; Zhang P. B.; Gu Q.; Leng Y. Catalytic Developments in the Epoxidation of Vegetable Oils and the Analysis Methods of Epoxidized Products. RSC Adv. 2019, 9 (65), 38119–38136. 10.1039/C9RA05943A. 35541772 PMC9075841

[ref5] Cogliano T.; Turco R.; Di Serio M.; Salmi T.; Tesser R.; Russo V. Epoxidation of Vegetable Oils via the Prilezhaev Reaction Method: A Review of the Transition from Batch to Continuous Processes. Ind. Eng. Chem. Res. 2024, 63 (26), 11231–11262. 10.1021/acs.iecr.3c04211.

[ref6] Meier M. A. R.; Metzger J. O.; Schubert U. S. Plant Oil Renewable Resources as Green Alternatives in Polymer Science. Chem. Soc. Rev. 2007, 36 (11), 1788–1802. 10.1039/b703294c. 18213986

[ref7] Chua S. C.; Xu X. B.; Guo Z. Emerging Sustainable Technology for Epoxidation Directed Toward Plant Oil-Based Plasticizers. Process Biochemistry 2012, 47 (10), 1439–1451. 10.1016/j.procbio.2012.05.025.

[ref8] Schwaiger M.; Resch-Fauster K. Mechanical Flexible Epoxy Resins with 100% Bio-Based Carbon Content Based on Epoxidized Vegetable Oils. J. Appl. Polym. Sci. 2022, 139 (48), e53233 10.1002/app.53233.

[ref9] Hood C.; Ghazani S. M.; Marangoni A. G.; Pensini E. Flexible Polymeric Biomaterials from Epoxidized Soybean Oil, Epoxidized Oleic Acid, and Citric Acid as Both a Hardener and Acid Catalyst. J. Appl. Polym. Sci. 2022, 139 (42), e53011 10.1002/app.53011.

[ref10] Cifarelli A.; Boggioni L.; Vignali A.; Tritto I.; Bertini F.; Losio S. Flexible Polyurethane Foams from Epoxidized Vegetable Oils and a Bio-Based Diisocyanate. Polymers 2021, 13 (4), 612 10.3390/polym13040612. 33670627 PMC7922077

[ref11] Meyer M.; Dietrich S.; Schulz H.; Mondschein A. Comparison of the Technical Performance of Leather, Artificial Leather, and Trendy Alternatives. Coatings 2021, 11 (2), 226 10.3390/coatings11020226.

[ref12] Zeng R. T.; Wu Y.; Li Y. D.; Wang M.; Zeng J. B. Curing Behavior of Epoxidized Soybean Oil with Biobased Dicarboxylic Acids. Polym. Test. 2017, 57, 281–287. 10.1016/j.polymertesting.2016.12.007.

[ref13] Di Mauro C.; Genua A.; Mija A. Fully Bio-Based Reprocessable Thermosetting Resins Based on Epoxidized Vegetable Oils Cured with Itaconic Acid. Industrial Crops and Products 2022, 185, 115116 10.1016/j.indcrop.2022.115116.

[ref14] Tellers J.; Jamali M.; Willems P.; Tjeerdsma B.; Sbirrazzuoli N.; Guigo N. Cross-Linking Behavior of Eutectic Hardeners from Natural Acid Mixtures. Green Chem. 2021, 23 (1), 536–545. 10.1039/D0GC03172K.

[ref15] Kadam A.; Pawar M.; Yemul O.; Thamke V.; Kodam K. Biodegradable Biobased Epoxy Resin from Karanja Oil. Polymer 2015, 72, 82–92. 10.1016/j.polymer.2015.07.002.

[ref16] Fustes-Damoc I.; Dinu R.; Malutan T.; Mija A. Valorisation of Chitosan Natural Building Block as a Primary Strategy for the Development of Sustainable Fully Bio-Based Epoxy Resins. Polymers 2023, 15 (24), 4627 10.3390/polym15244627. 38139881 PMC10747223

[ref17] Supanchaiyamat N.; Shuttleworth P. S.; Hunt A. J.; Clark J. H.; Matharu A. S. Thermosetting Resin Based on Epoxidised Linseed Oil and Bio-Derived Crosslinker. Green Chem. 2012, 14 (6), 1759–1765. 10.1039/c2gc35154d.

[ref18] Ding C.; Shuttleworth P. S.; Makin S.; Clark J. H.; Matharu A. S. New Insights into the Curing of Epoxidized Linseed Oil with Dicarboxylic Acids. Green Chem. 2015, 17 (7), 4000–4008. 10.1039/C5GC00912J.

[ref19] Di Mauro C.; Tran T. N.; Graillot A.; Mija A. Enhancing the Recyclability of a Vegetable Oil-Based Epoxy Thermoset through Initiator Influence. ACS Sustainable Chem. Eng. 2020, 8 (20), 7690–7700. 10.1021/acssuschemeng.0c01419.

[ref20] Meyer M.; Dietrich S.; Schulz H.; Mondschein A. Comparison of the Technical Performance of Leather, Artificial Leather, and Trendy Alternatives. Coatings 2021, 11 (2), 226 10.3390/coatings11020226.

[ref21] Wells H. C.; Edmonds R. L.; Kirby N.; Hawley A.; Mudie S. T.; Haverkamp R. G. Collagen Fibril Diameter and Leather Strength. J. Agric. Food Chem. 2013, 61 (47), 11524–11531. 10.1021/jf4041854. 24199635

[ref22] Prajapati H.; Tevatia A.; Dixit A. Advances in Natural-Fiber-Reinforced Composites: A Topical Review. Mechanics of Composite Materials 2022, 58 (3), 319–354. 10.1007/s11029-022-10033-2.

[ref23] Bledzki A. K.; Urbaniak M.; Boettcher A.; Berger C.; Pilawka R. Bio-Based Epoxies and Composites for Technical Applications. Key Engineering Materials 2013, 559, 1–6. 10.4028/www.scientific.net/KEM.559.1.

[ref24] Wang Z.; Zhang X.; Wang R.; Kang H.; Qiao B.; Ma J.; Zhang L.; Wang H. Synthesis and Characterization of Novel Soybean-Oil-Based Elastomers with Favorable Processability and Tunable Properties. Macromolecules 2012, 45 (22), 9010–9019. 10.1021/ma301938a.

[ref25] Intarabumrung W.; Kuntharin S.; Harnchana V.; Prada T.; Kasemsiri P.; Hunt A. J.; Supanchaiyamat N. Facile Synthesis of Biobased Polyamide Derived from Epoxidized Soybean Oil as a High-Efficiency Triboelectric Nanogenerator. ACS Sustainable Chem. Eng. 2022, 10 (41), 13680–13691. 10.1021/acssuschemeng.2c03592.

[ref26] Di Mauro C.; Malburet S.; Genua A.; Graillot A.; Mija A. Sustainable Series of New Epoxidized Vegetable Oil-Based Thermosets with Chemical Recycling Properties. Biomacromolecules 2020, 21 (9), 3923–3935. 10.1021/acs.biomac.0c01059. 32790997

[ref27] Wang T. T.; Ye P.; Xu X.; Lu M. Q.; Zhang X. Y.; Li N. Q. Metabolic Engineering Combined with Site-Directed Saturated Mutations of α-Keto Acid Decarboxylase for Efficient Production of 6-Aminocaproic Acid and 1,6-Hexamethylenediamine. Biotechnol. Bioeng. 2024, 121, 3329–3337. 10.1002/bit.28795. 38956978

[ref28] Dros A. B.; Larue O.; Reimond A.; De Campo F.; Pera-Titus M. Hexamethylenediamine (HMDA) from Fossil- vs. Bio-Based Routes: An Economic and Life Cycle Assessment Comparative Study. Green Chem. 2015, 17 (10), 4760–4772. 10.1039/C5GC01549A.

[ref29] Covestro und Genomatica Produzieren Wichtigen Chemierohstoff mit Biotechnologie. https://www.covestro.com/press/de/covestro-und-genomatica-produzieren-wichtigen-chemierohstoff-mit-biotechnologie/ (accessed Oct 17, 2024).

[ref30] Shechter L.; Wynstra J. Glycidyl Ether Reactions with Alcohols, Phenols, Carboxylic Acids, and Acid Anhydrides. Ind. Eng. Chem. 1956, 48 (1), 86–93. 10.1021/ie50553a028.

[ref31] Li Y.; Xiao F.; Wong C. P. Novel, Environmentally Friendly Crosslinking System of an Epoxy Using an Amino Acid: Tryptophan-Cured Diglycidyl Ether of Bisphenol A Epoxy. J. Polym. Sci., Part A: Polym. Chem. 2007, 45 (2), 181–190. 10.1002/pola.21742.

[ref32] Gobin M.; Loulergue P.; Audic J. L.; Lemiègre L. Synthesis and Characterisation of Bio-Based Polyester Materials from Vegetable Oil and Short to Long Chain Dicarboxylic Acids. Industrial Crops and Products 2015, 70, 213–220. 10.1016/j.indcrop.2015.03.041.

[ref33] Ding X. M.; Chen L.; Guo D. M.; Liu B. W.; Luo X.; Lei Y. F.; Zhong H. Y.; Wang Y. Z. Controlling Cross-Linking Networks with Different Imidazole Accelerators Toward High-Performance Epoxidized Soybean Oil-Based Thermosets. ACS Sustainable Chem. Eng. 2021, 9 (8), 3267–3277. 10.1021/acssuschemeng.0c08852.

[ref34] Ahn K.; Zaccaron S.; Zwirchmayr N. S.; Hettegger H.; Hofinger A.; Bacher M.; Henniges U.; Hosoya T.; Potthast A.; Rosenau T. Yellowing and Brightness Reversion of Celluloses: CO or COOH, Who Is the Culprit?. Cellulose 2019, 26 (1), 429–444. 10.1007/s10570-018-2200-x.

[ref35] Korntner P.; Hosoya T.; Dietz T.; Eibinger K.; Reiter H.; Spitzbart M.; Roeder T.; Borgards A.; Kreiner W.; Mahler A. K.; Winter H.; Groiss Y.; French A. D.; Henniges U.; Potthast A.; Rosenau T. Chromophores in Lignin-Free Cellulosic Materials Belong to Three Compound Classes. Chromophores in Cellulosics, XII. Cellulose 2015, 22 (2), 1053–1062. 10.1007/s10570-015-0566-6.

