# Supplementary material for: Flexible Biobased Thermosets from Epoxidized Plant Oils: A Study of Aliphatic Cross-Linking Agents
Source: ACS Appl Polym Mater. 2025 Mar 12;7(6):3686–97. doi: 10.1021/acsapm.4c03944 (PMC11959707; doi:10.1021/acsapm.4c03944)
Supplement: Supplementary file 1 — ap4c03944_si_001.pdf [file ap4c03944_si_001.pdf]

Supporting Information

Flexible biobased thermosets from epoxidized plant oils: A study of aliphatic cross-linking agents

Jan Janesch*[1,2], Axel Solt-Rindler[3], Lara Dumschat[2,3], Oliver Vay[3], Alice Mija[4], Wolfgang Gindl-Altmutter[1], Thomas Rosenau[2], Wolfgang Raffeiner[5], Christian Hansmann[1,3]

1 Institute of Wood Technology and Renewable Materials, Department of Natural Sciences and Sustainable Resources, BOKU - University of Natural Resources and Life Sciences, Vienna, Konrad-Lorenz-Straße 24, 3430 Tulln, Austria.

2 Institute of Chemistry of Renewable Resources, Department of Natural Sciences and Sustainable Resources, BOKU - University of Natural Resources and Life Sciences, Vienna, Konrad-Lorenz-Straße 24, 3430 Tulln, Austria.

3 Wood K plus-Competence Centre for Wood Composites & Wood Chemistry, Kompetenzzentrum Holz GmbH, Altenberger Straße 69, 4040 Linz, Austria.

4 Université Côte d'Azur, Institute of Chemistry of Nice, UMR CNRS 7272, 06108 Nice CEDEX 2, France

5 Sozialgenossenschaft „Lebenswertes Ulten", Gewerbezone Schmiedhof 349, 39016 St. Walburg, Italy

*Corresponding author contact details: jan.janesch@boku.ac.at

Contents:

Number of pages: 4

Number of tables: 0

Number of figures: 5

1. Curing Enthalpy

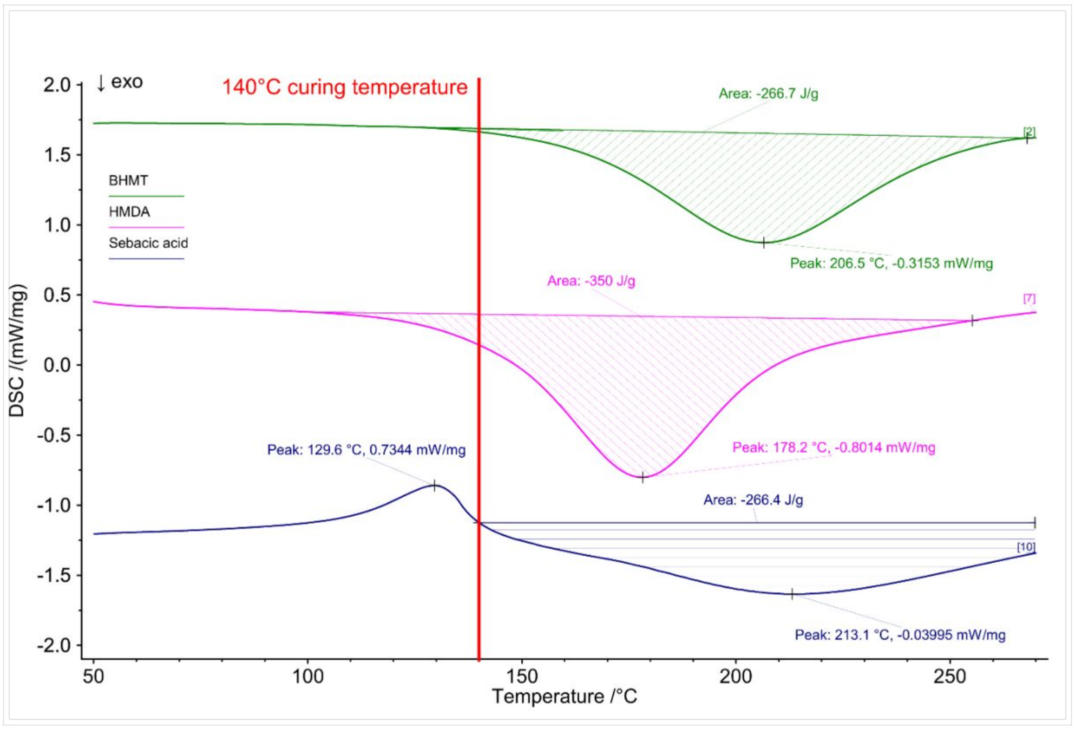

Figure S1: Differential Scanning Calorimetry of ELO mixed with the 3 hardeners.

2. Elastic modulus and normalized elongation at break

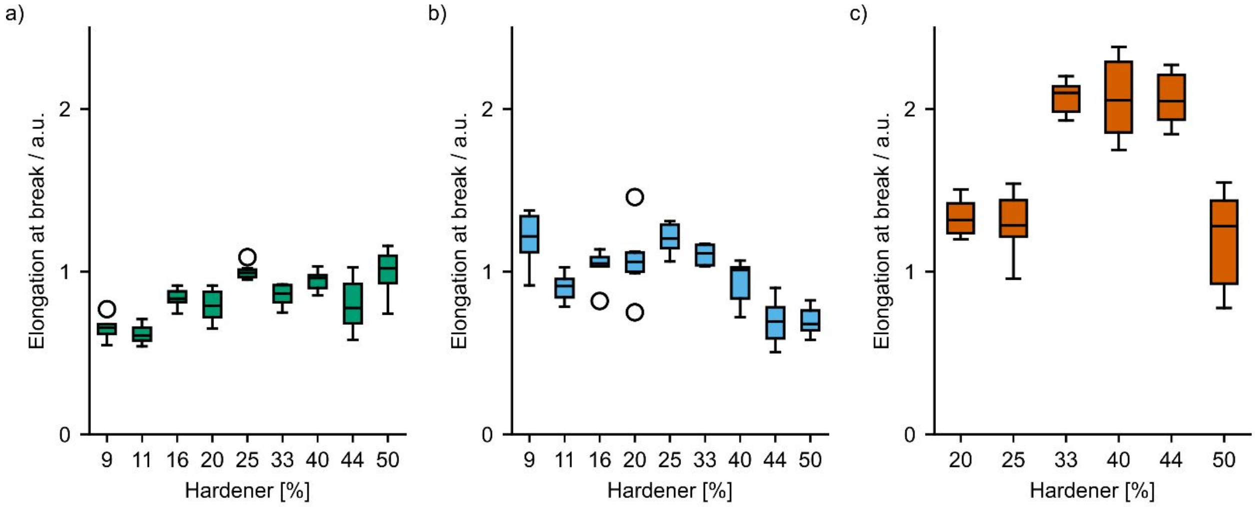

Figure S2: Elongation at break for ELO cured with sebacic acid (a), bis(hexamethylene)triamine (b), and hexamethylenediamine (c), normalized to the mean of ELO cured with 25% sebacic acid.

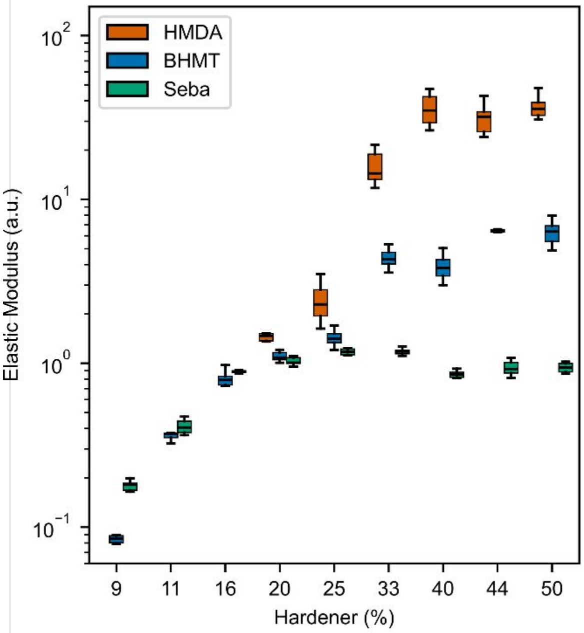

Figure S3: Elastic modulus for ELO cured with three types of hardeners.

3. Raman spectroscopy

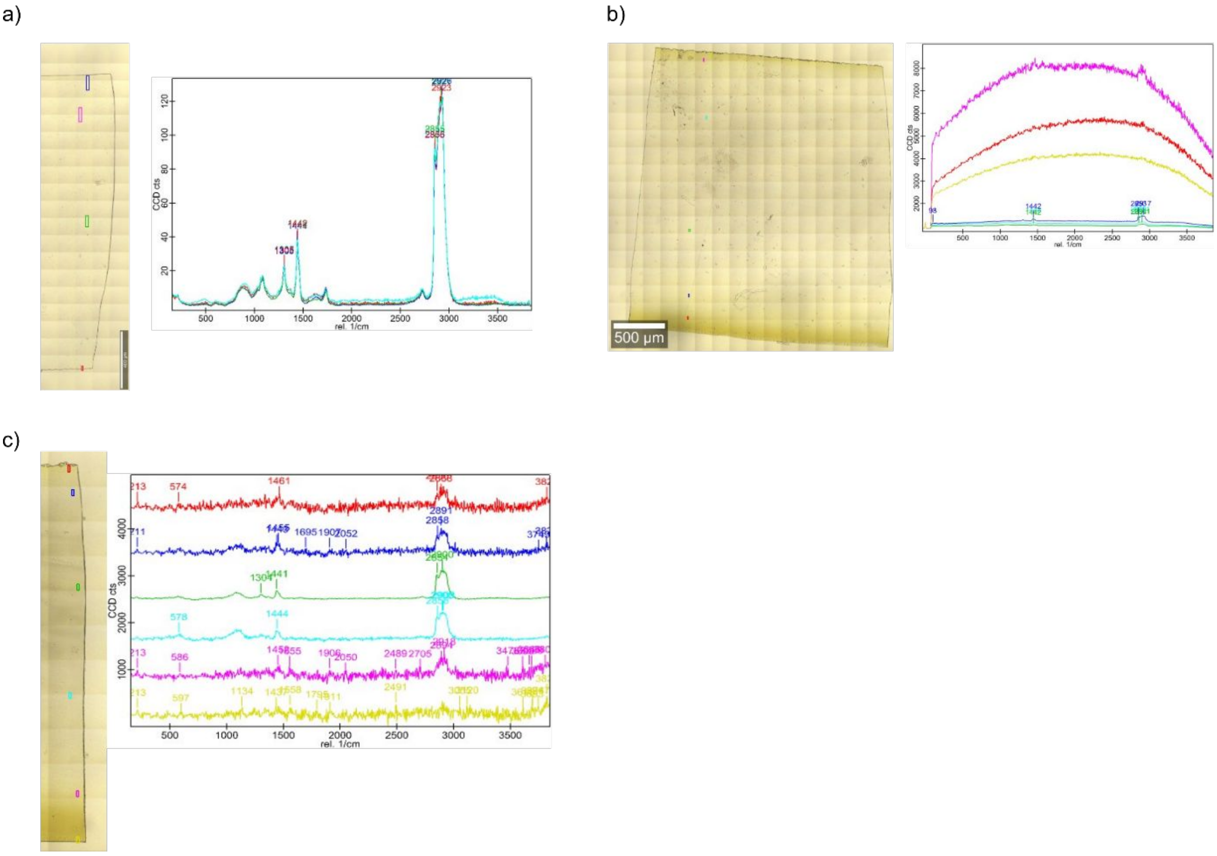

Figure S4: Raman-spectroscopy on cut cross-sections of Elo cured with sebacic acid (a), BHMT (b), and HMDA (c).

4. FT-IR spectra of thermoset samples

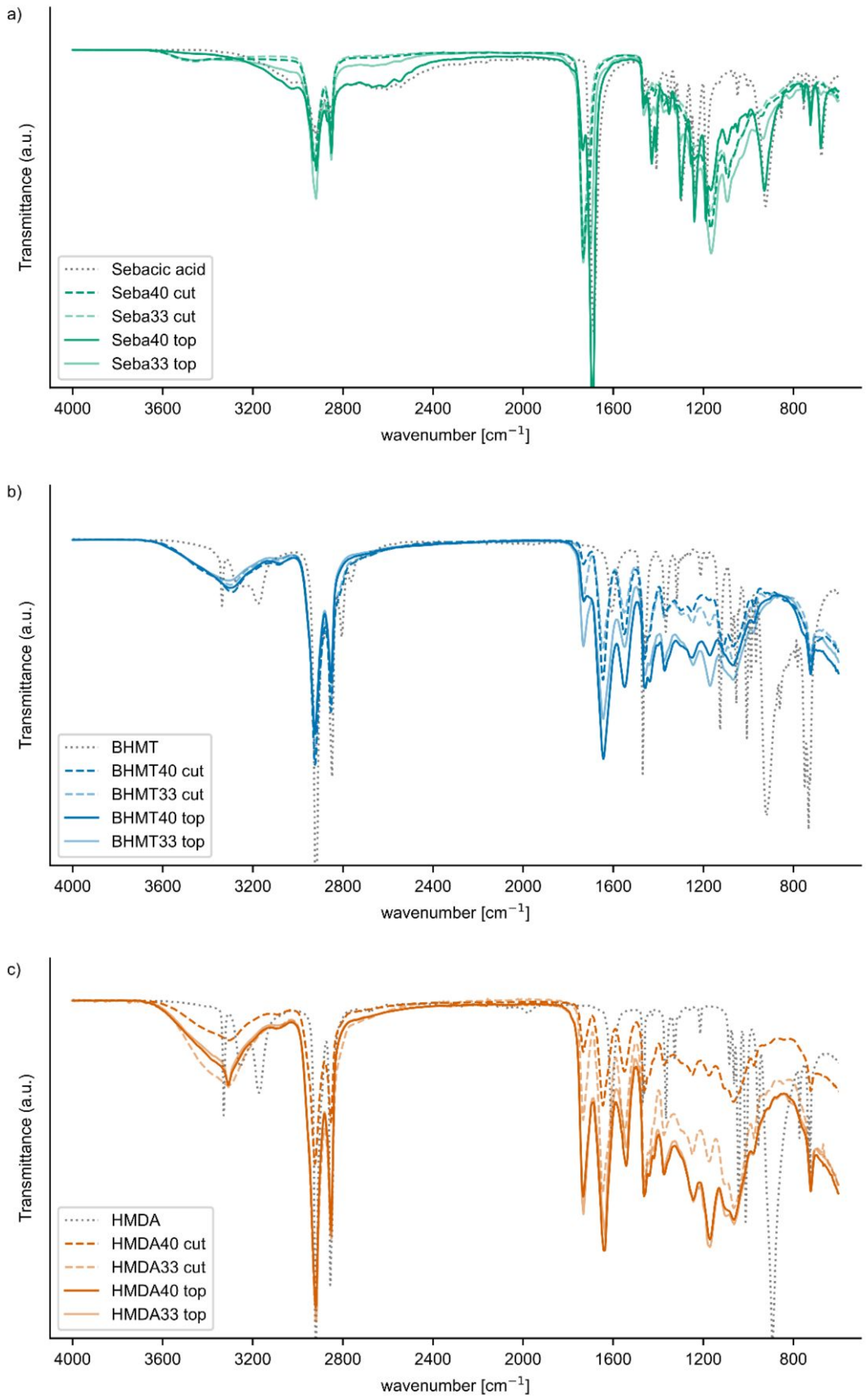

Figure S5: FTIR spectra of ELO cured with sebacic acid (a), BHMT (b), and HMDA (c). Dashed lines are spectra from inner surfaces of the sample, while solid lines represent outer surfaces.
